# Investigating the causal relationship between immune factors and ankylosing spondylitis: insights from a Mendelian Randomization study

**DOI:** 10.1186/s42358-024-00428-1

**Published:** 2024-12-18

**Authors:** Ziming Geng, Yang Tong, Yang Chen, Jian Wang, Ziwen Liu, Jun Miao, Ruihua Li

**Affiliations:** 1 Department of Orthopedics, Chinese PLA General Hospital, Beijing, 100141 China; 2 School of Finance, Nankai University, Tianjin, 300350 China; 3 Academy of Medical Engineering and Translational Medicine, Tianjin University, Tianjin, 300072 China; 4 Tianjin Hospital, Tianjin University, No. 406 Jiefang South Rd, Hexi District, Tianjin, 300211 China; 5 Department of Spine Surgery, Tianjin Hospital, Tianjin University, No. 406 Jiefang South Rd, Hexi District, Tianjin, 300211 China

**Keywords:** Ankylosing spondylitis, Immunity, Immune cells, Mendelian Randomization, FinnGen

## Abstract

**Background:**

Despite previous studies indicating a close relationship between immune system and ankylosing spondylitis (AS), the causal relationship between them remains unclear.

**Methods:**

Genome-wide association data were utilized to explore the causal link between 731 immune cells and AS using a bidirectional two-sample MR approach. The data included immune cell data from Orrù et al.’s study and AS data from the FinnGen consortium. Cochran’s Q test and leave-one-out checked instrument variable (IV) heterogeneity. IVW was the primary method for causal analysis, with MR-Egger and MR-PRESSO addressing horizontal pleiotropy. FDR correction was applied to both analysis directions to rectify multiple testing errors.

**Results:**

In our study, 22 immune phenotypes out of 731 were casually linked to AS. After excluding 5 less robust features, 17 immune factors remained, with 4 being protective and the rest posing risks. Through FDR correction, we found a significant causal relationship between HLA DR on CD14- CD16+ monocyte and AS (OR (95%CI) = 0.70(0.60 ~ 0.83), *P* = 2.06*10^−5^). In the reverse analysis with AS as exposure, potential effects on 34 immune features were discovered. After correction, we confirmed significant causal relationships between AS and two immune features, namely CD20- B cell %lymphocyte (OR (95%CI) = 1.16(1.08–1.25), *P* = 1.91*10^−5^) and CD20- B cell %B cell (OR (95%CI) = 1.17(1.09–1.26), *P* = 1.50*10^−5^).

**Conclusions:**

Our study identified various features associated with AS in different types of immune cells. These findings provide important clues and a theoretical basis for further understanding the pathogenesis of AS, guiding clinical treatment, and drug design.

**Supplementary information:**

The online version contains supplementary material available at 10.1186/s42358-024-00428-1.

## Introduction

Ankylosing spondylitis (AS) is a chronic inflammatory joint disease characterized by immune-mediated inflammation affecting axial skeleton, including spine, sacroiliac joints, and entheses. It leads to chronic back pain, progressive loss of joint function, and spinal mobility impairment, often accompanied by complications involving extraspinal organs [1, 2]. Approximately 0.32 to 1.4% of the population is affected by AS, a condition that typically manifests in individuals over the age of 20, with a male-to-female ratio of 2:1 [3, 4]. AS can lead to long-term disability, and patients with AS have a higher mortality rate than the general population, imposing a significant societal burden [5]. The pathogenesis of AS is complex and multifactorial, involving environmental, genetic, and immunological factors [6, 7]. Immunological disturbances, in particular, can trigger or contribute to the onset of AS. Even while anti-inflammatory treatments for AS have made significant progress, the underlying mechanisms remain mostly unknown.

In recent years, evidence has gradually revealed the close relationship between the immune system and the skeletal system, where the skeletal system impacts the immune system, and correspondingly, various immune cells and cytokines are involved in the pathological processes of bone homeostasis and inflammatory orthopedic diseases [8, 9]. Innate cells have been implicated in the pathophysiology of AS, including neutrophils, mast cells, macrophages, as well as innate lymphoid cells and innate-like T cells [2, 10], highlighting the crucial role of immune factors in the mechanisms underlying AS. In individuals with ankylosing spondylitis (AS), the proportion of CD8+ CD122+ T cells in peripheral blood is higher compared to healthy controls [11]. Additionally, there is a reduction in CD27+ B cells, while the numbers of CD86+ and CD27-CD95+ B cells are increased [12]. These findings suggest that these immune cells may play a role in the pathogenesis and progression of AS. Following the identification of a significant correlation between ankylosing spondylitis (AS) and the human leukocyte antigen HLA-B27 [13], subsequent studies confirmed its presence in up to 90% of patients in most ethnic populations affected by the disease [14]. GWAS results also indicated the involvement of both adaptive and innate immune systems, with TNF and IL-23 pathways being the major effector pathways [15]. This dysregulation accompanies the development of immune imbalance. However, while these findings confirm the intimate connection between AS and the immune system, it is still unclear which particular immunological components are responsible for this relationship.

Mendelian Randomization (MR) is an innovative research approach that utilizes Single Nucleotide Polymorphisms (SNPs) as instrumental variables (IVs) to infer causal relationships between exposure and outcomes. Since genetic variations occur at conception and are nearly unaffected by outcomes, they are not influenced by confounding factors or reverse causation. Compared to traditional epidemiological studies, MR significantly enhances statistical power and result reliability [16]. In this study, we employed bidirectional two-sample MR to investigate the associations between immune cell phenotypes in peripheral blood, such as T cell maturation stages, TBNK cells, Treg cells, B cells, Myeloid cells, conventional dendritic cells (cDC), and monocytes, and AS. This exploration aims to elucidate the relationship between immune features and the onset of AS, providing theoretical foundations and guiding principles for early diagnosis and treatment of AS.

## Materials and methods

### Study design

Based on a comprehensive summary-level dataset derived from large-scale Genome-Wide Association Study (GWAS) data, we employed a bidirectional two-sample Mendelian Randomization approach to assess the causal relationships between a vast array of immune cells (comprising 731 varieties from 7 immune panels and 4 phenotypic categories) and the risk of Ankylosing Spondylitis. All studies included in the dataset had received approval from relevant institutional review boards, and participants provided informed consent. Conducting this research did not require additional informed consent.

### Data source

The data on immune cells were obtained from a study conducted by Orrù et al. [17]. 118 absolute cell counts (AC), 32 morphological parameters (MP), 389 median fluorescence intensities (MFI) representing surface antigen levels, and 192 relative cell counts (RC) were all analyzed in this study using flow cytometry. A total of 731 immune phenotypes were assessed in a normal population consisting of 3757 individuals from Sardinia. Genotyping of the samples was performed using four Illumina arrays (OmniExpress, ImmunoChip, Cardio-MetaboChip, and ExomeChip), and whole-genome imputation was carried out based on a reference panel of 3514 individuals from the Sardinian sequence. Approximately 22 million variants were retained for the association analysis after adjusting for covariates such as gender, age, and age squared [18]. Ultimately, 122 significant independent association signals were detected at 70 loci (including 53 novel loci) out of 459 cellular traits (*P* < 1.28 × 10^−11^), elucidating multiple molecules and mechanisms involved in cell regulation. Summary statistics for a total of 731 immune features were downloaded from the GWAS catalog (accession numbers from GCST90001391 to GCST90002121). Detailed information about the panels and phenotype categories for each immune phenotype can be found in Supplementary Table 1.

GWAS summary data for AS were acquired from the FinnGen Consortium R9 release [19], which included 2860 cases and 389,563 controls. The diagnosis of AS was based on ICD-9 7200, ICD-10 M45 and ICD-8 7124. Table 1 provides comprehensive details about the outcome and exposure examined in this investigation.

**Table 1:** Details of the exposure and outcome

Trait	Year	Consortium/Author	Samples	Case	Control	Sex
731 immune phenotypes	2020	Orrù et al	3757	/	/	57.0% female
Ankylosing spondylitis	2023	FinnGen (R9)	392,423	2860	389,563	Mixed

### Instrumental variable selection

Effective Instrumental Variables (IVs) need to fulfill three assumptions: (1) relevance: IVs should have a relationship with the exposure; (2) independence: IVs should not be influenced by extraneous variables; and (3) exclusion restriction: IVs must be conditionally independent of the outcomes in light of the exposure.

We employed the PLINK software’s clumping approach, setting a significance threshold of 1 × 10^−5^, to extract independent and significant SNPs for each immunological characteristic, drawing on prior research [17, 20, 21]. Based on the 1000 Genomes Project reference panel, the Linkage Disequilibrium (LD) r^2^ threshold was determined to be less than 0.1 within a 500 kb radius. Linkage Disequilibrium (LD) r^2^ threshold was set to be less than 0.1 within a distance of 500 kb, calculated based on the 1000 Genomes Project reference panel [22]. For AS, a more stringent threshold was set, with a significance threshold of 5.00 × 10^−8^ and r^2^ of 0.01. We calculated the fraction of phenotypic variation explained (R^2^) and evaluated the strength of instrumental variables (IVs) for each immunological characteristic using the F statistic in order to reduce the impact of weak instrumental bias in MR analysis. IVs with F statistics below 10 were excluded from our analysis. We identified 7–1786 independent IVs associated with 731 immune phenotypes, with these selected IVs explaining an average of 0.240% of the variance in their respective phenotypes (ranging from 0.004 to 3.652%). Detailed information about all positive result SNPs can be found in Supplementary Table 2.

### Mendelian Randomization analysis

In Mendelian Randomization (MR) analysis, when the core assumptions are met, the Inverse Variance Weighted (IVW) method is employed to improve statistical power and enhance the accuracy of estimates [23]. So the IVW method functions as the primary approach to assess the overall causal relationship between immune traits and Ankylosing Spondylitis (AS). However, in the presence of horizontal pleiotropy, causal estimates using the IVW method might be biased. Consequently, genetic variations could influence susceptibility to AS through pathways outside immune traits. We employed the MR-Egger regression method [24] to assess whether the intercept significantly deviated from the origin and used the Mendelian Randomization Pleiotropy Residual Sum and Outlier (MR-PRESSO) to identify the existence of pleiotropy [25]. All positive outcomes derived from the Mendelian randomization analysis are categorized based on panels and presented in the form of a heatmap.

Supplementary methods including MR-Egger, Weighted Median, Weighted Mode, and Simple Mode were employed. Cochran’s Q test and leave-one-out analysis were performed to assess the heterogeneity of IVs. Reverse analysis was utilized to explore whether AS causally impacts immune traits, ensuring the unidirectionality of causality. To avoid false positives due to multiple testing, FDR correction was applied to both directions of the analysis.

## Results

**Fig. 1:**
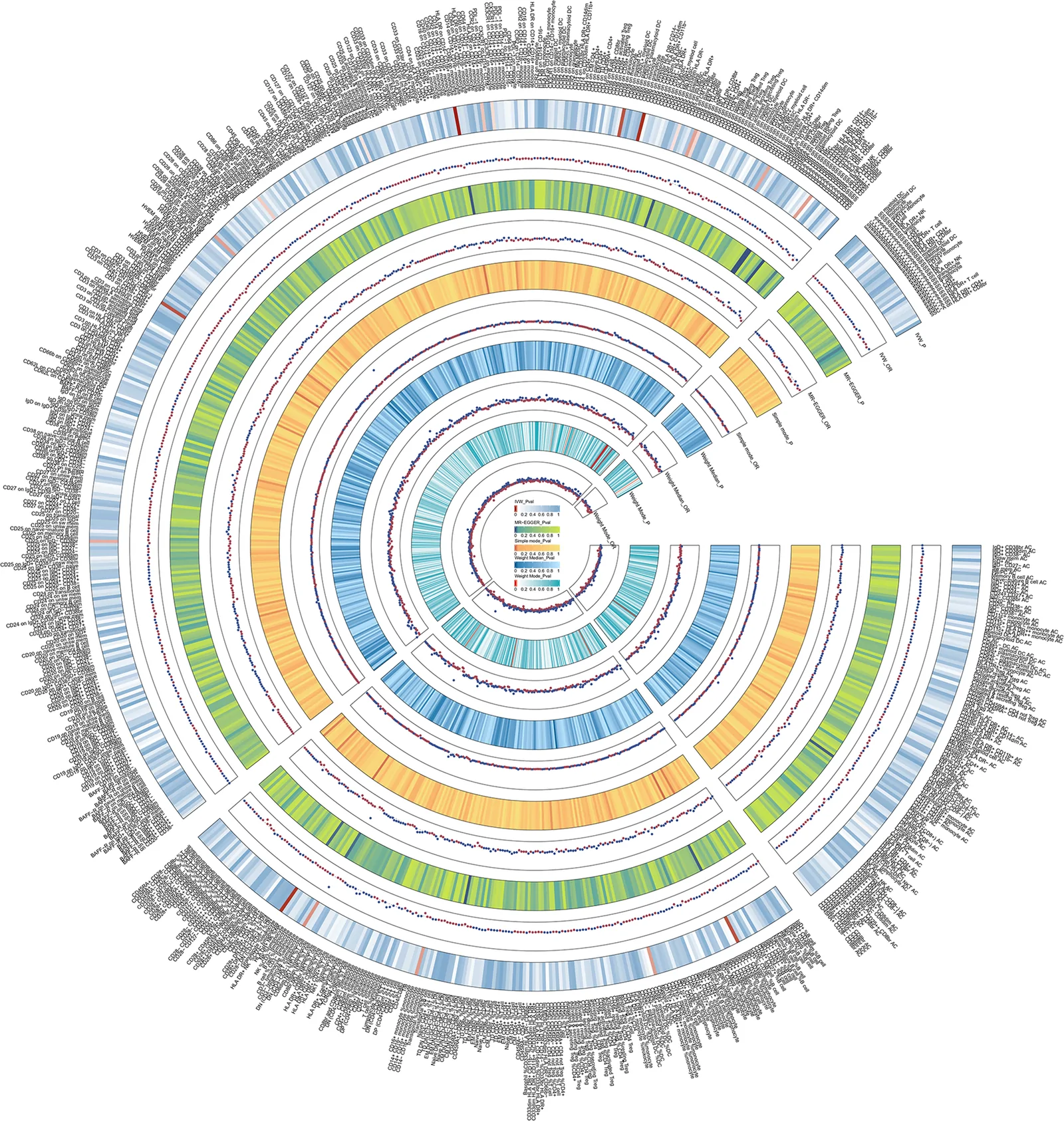
Circular heatmap for Mendelian Randomization analysis result: impact of immune cells on the risk of ankylosing spondylitis (AS)

Through Mendelian Randomization analysis, a total of 22 *nominally* significant causal associations (*P* < 0.05) were identified between 731 immune features and Ankylosing Spondylitis (AS) (Fig. 1). Considering the immune features were categorized into four phenotypes (AC, MFI, RC, and MP) across seven immune panels, the potential immune features associated with AS included 1 AC, 16 MFI, and 5 RC traits. After classifying immune traits based on the seven panels, two characteristics were discovered to correspond to the T cell maturation stages, three to TBNK cells, four to Treg cells, three to B cells, three to Myeloid cells, three to cDC, and four to monocytes (Supplementary Figure 1A). These traits showed suggestive associations with AS. Among them, 5 phenotypes/AS associations were found to be non-robust in further analysis and were excluded (inconsistent directions across five analytical methods). Among the remaining 17 immune features, 4 were protective factors, while the rest were risk factors (Supplementary Table 3). Considering the possibility of false positives, we applied FDR correction to *P*-values for different types of traits. After multiple testing correction and sensitivity analysis, only one immune phenotype was found to have a causal association with AS (FDR-adjusted *P* < 0.05). Specifically, HLA DR on CD14- CD16+ monocyte1 (OR (95%CI): 0.70 (0.60–0.83), *P* = 2.06*10^−5^, *P* (FDR) = 1.51*10^−2^) (Supplementary Figure 2).

**Fig. 2:**
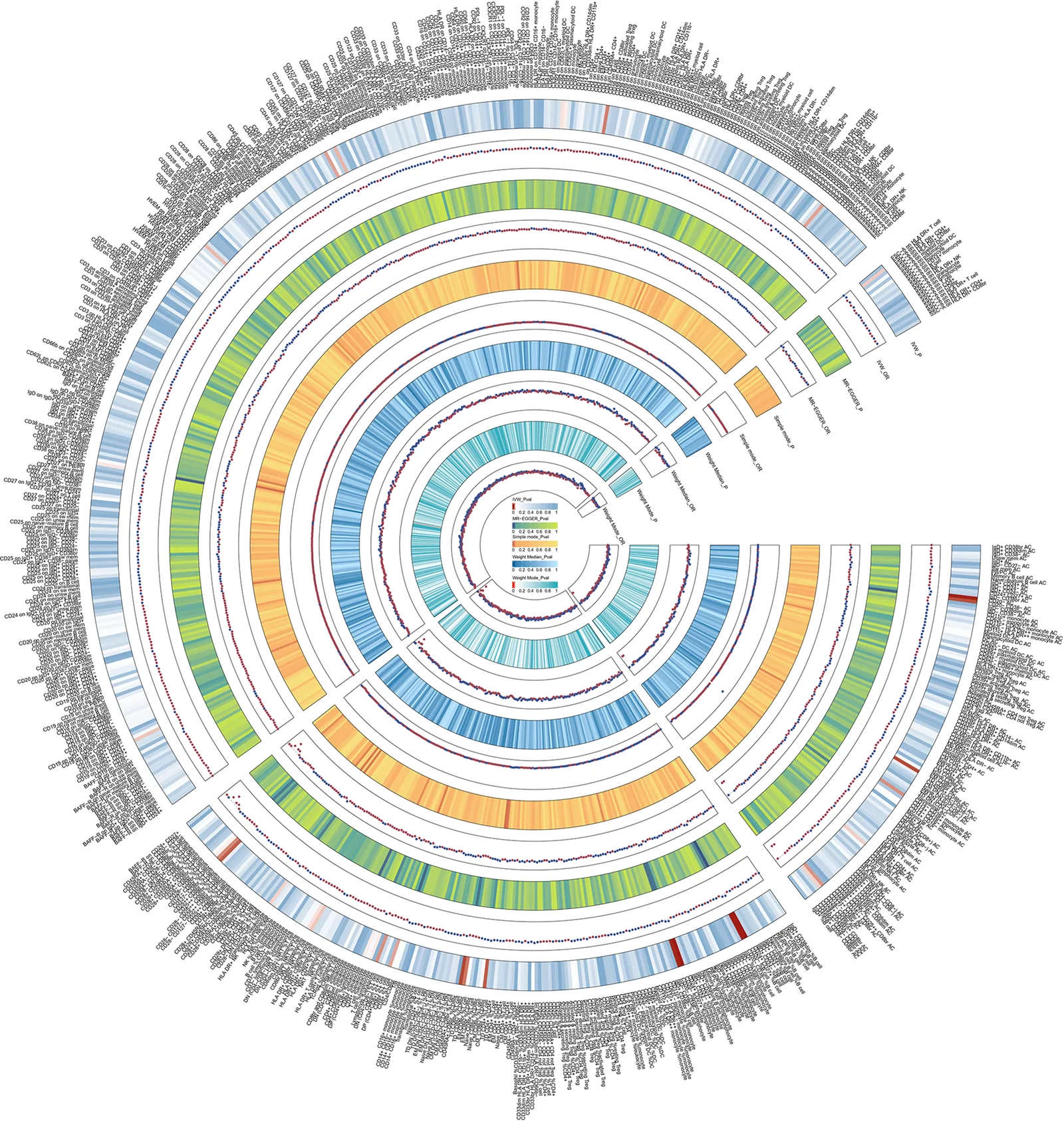
Circular heatmap for Mendelian Randomization analysis result: impact of ankylosing spondylitis on immune cells

To comprehend how the body’s immunological processes are impacted by AS development, We performed MR analysis to investigate the causative effect of Ankylosing Spondylitis (AS) on immune cells. We used AS as the exposure and 731 immune features as outcomes for reverse analysis. Out of the 12 sets of analyses, which couldn’t be performed due to the lack of sufficient instrumental variables, we found *nominal* effects of AS on 34 immune features (*P* < 0.05) (Fig. 2). Similarly, we elaborated the results based on phenotype types and immune cell grouping (Supplementary Figure 1B): these effects comprised 7 AC, 17 RC, 1 MP, and 9 MFI; corresponding panel classifications included 5 traits related to the maturation stages of T cells, 2 to cDC, 7 to Treg, 1 to monocytes, 8 to B cells, 3 to Myeloid cells, and 11 to TBNK. AS was suggestive of being correlated with these traits. Among them, 4 AS/phenotype pairs were found to be non-robust in sensitivity analysis and were excluded. Among the remaining 30 immune phenotypes, AS was negatively correlated with 19 of them and positively associated with the left (Supplementary Table 4). After FDR correction for reverse analysis, AS remained significantly causally associated with two immune phenotypes: CD20- B cell %lymphocyte (OR (95%CI): 1.16 (1.08–1.25), *P* = 1.91*10^−5^, *P* (FDR) = 6.87*10^−3^) and CD20- B cell %B cell (OR (95%CI): 1.17 (1.09–1.26), *P* = 1.50*10^−5^, *P* (FDR) = 6.87*10^−3^). The scatter plot (Supplementary Figure 2) indicates a positive correlation between these two features and the onset of AS.

In summary, significant associations with AS were observed, whether based on the four main types of immune features or the seven subgroups of immune cells. The heterogeneity and pleiotropy analysis results for positive outcomes in the bidirectional analysis are presented in Supplementary Tables 5 and 6, respectively. Here, we summarized the complex results into four sections based on immune phenotype types.

### Absolute cell counts and AS

In this phenotype classification, HLA DR+ CD4+ AC is a risk factor for the onset of AS (OR (95%CI): 1.14 (1.00–1.30), *P* = 0.048). Cochran’s Q test revealed some heterogeneity in the IVs (Supplementary Table 5). Nevertheless, the MR-Egger’s intercept showed no significant deviation from zero, indicating the absence of horizontal pleiotropy, suggesting no apparent horizontal pleiotropy. Additionally, the MR-PRESSO analysis did not identify any potential instrumental variable outliers at a significance level of 0.05.

**Fig. 3:**
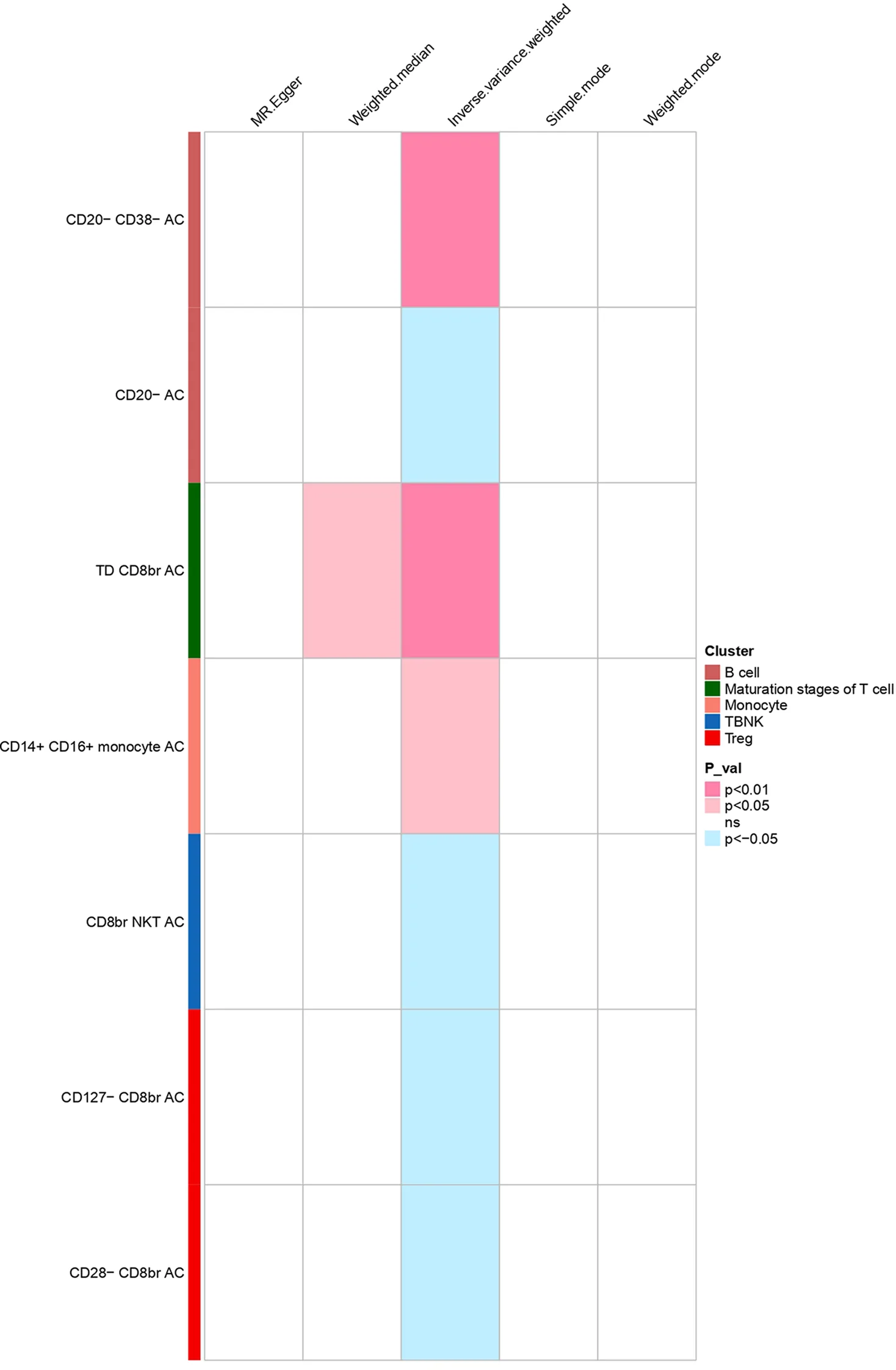
Mendelian randomization associations of AS on immune traits (AC)

In the reverse MR analysis, the onset of AS was found to be associated with 7 immune traits distributed across five panels: B cell, Maturation stages of T cell, Monocyte, Treg, and TBNK (Fig. 3). Among them, CD20- B cell and Terminally Differentiated CD8+ T cell showed the highest significance, with *p*-values of 1.197*10^−3^ and 8.407*10^−3^, respectively. After FDR correction, no AC traits were found to be significantly associated with the risk of AS.

### Relative cell counts and AS

**Fig. 4:**
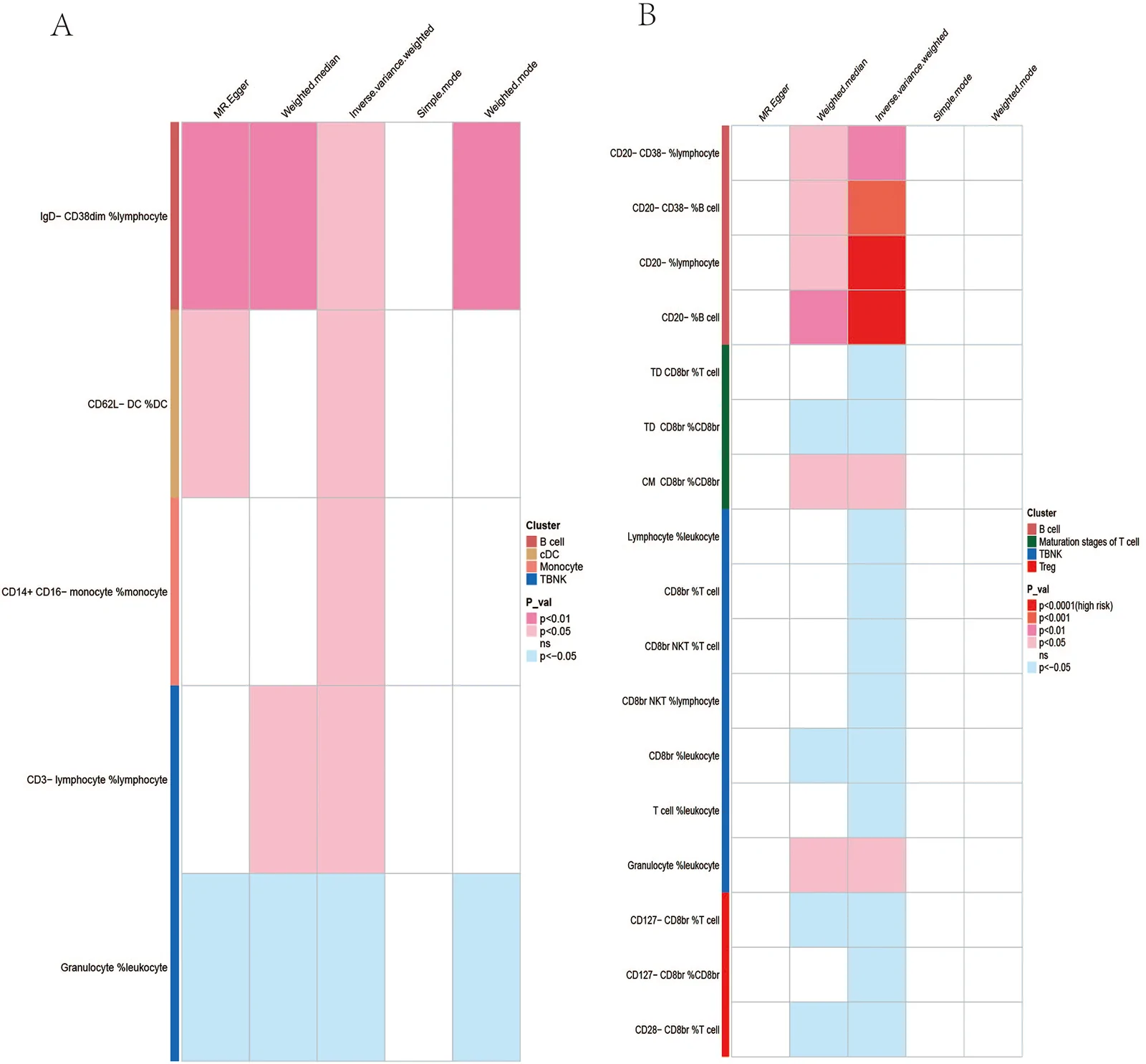
Mendelian randomization associations between AS and immune traits (RC). (**A**) The forward MR analysis; (**B**) The reverse MR analysis

Five RC traits/AS connections in total were found in the forward MR analysis, as indicated in Fig. 4A, and they all reached a significance level of *P* < 0.05. Among them, CD3- lymphocyte %lymphocyte showed inconsistent direction with IVW in sensitivity analysis. The remaining four, except Granulocyte %leukocyte, were risk factors for the onset of AS.

In the reverse MR analysis, we identified 17 nominally significant RC traits/AS associations (Fig. 4B). Of them, the start of AS caused 11 immune cell levels to drop and 6 immune cell levels to rise. Of these, the onset of AS caused an increase in 6 immune cells and a decrease in 11 immune cells. These 17 immune cells were split up into four panels: three for T cells, four for B cells, seven for TBNK, and three for Treg. After multiple testing adjustment, we found two immune cells with causal relationships achieving FDR significance (FDR < 0.05): CD20- B cell %B cell (*P* = 1.51*10^−5^, *P*(FDR) = 6.87*10^−3^) and CD20- B cell %lymphocyte (*P* = 1.91*10^−5^, *P*(FDR) = 6.87*10^−3^), both within the B cell panel. This suggests that the onset of AS increases the levels of these two immune cells.

### Morphological parameter and AS

No immune phenotype was found to be associated with the onset of AS in the forward analysis. However, in the reverse analysis, a causal relationship was identified between the onset of AS and FSC-A on Natural Killer T, a morphological parameter. AS onset was associated with an increase in the volume of Natural Killer T cells (OR (95%CI) = 1.08(1.00–1.17), *P* = 0.03), indicating the potential significant role of these cells in the AS disease process.

### Median fluorescence intensities and AS

**Fig. 5:**
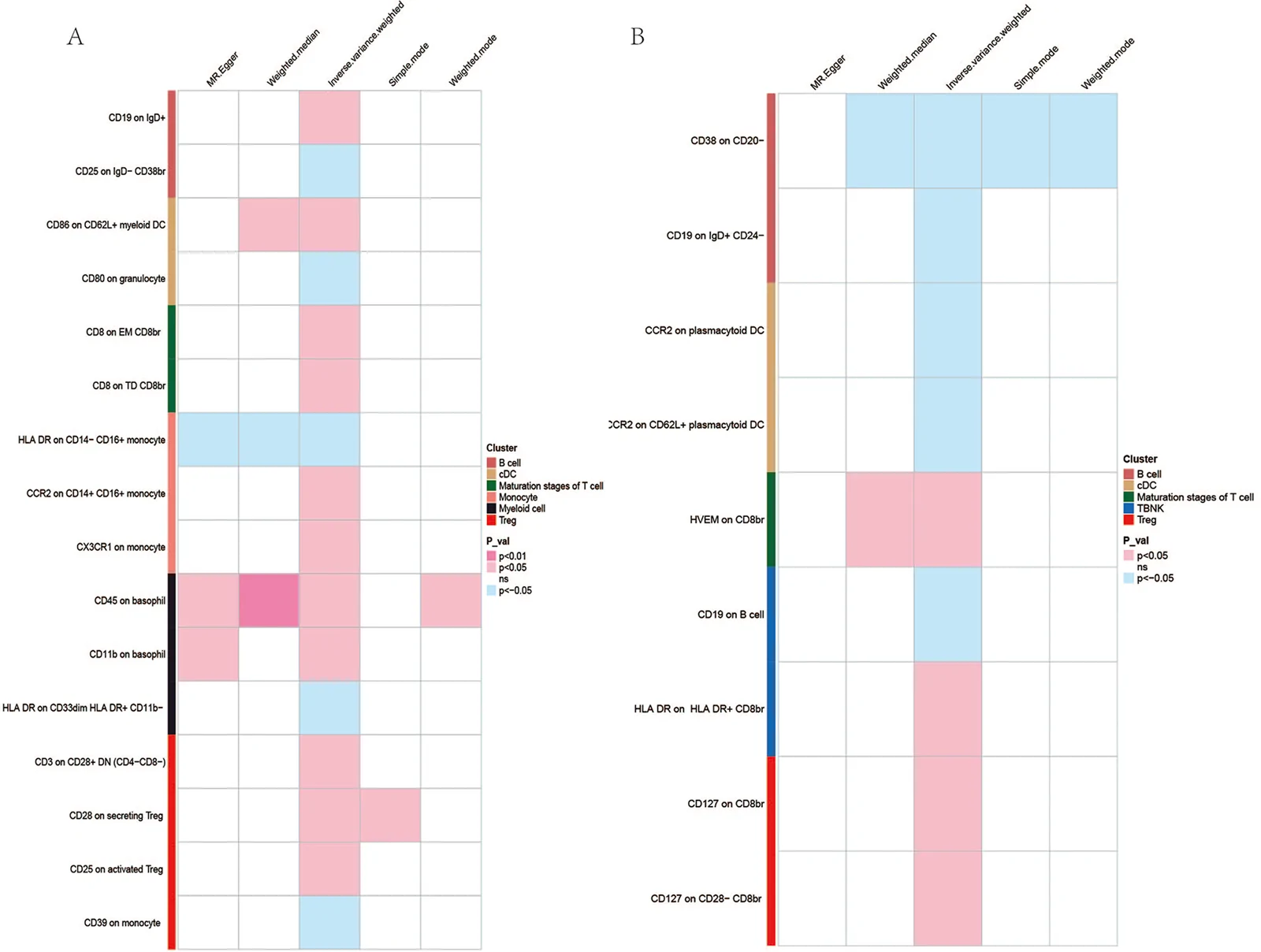
Mendelian randomization associations between AS and immune traits (MFI). (**A**) The forward MR analysis; (**B**) The reverse MR analysis

Figure 5A displays 16 suggestive associations (*P* < 0.05) between immune phenotypes and AS. Among them, HLA DR on CD14- CD16+ monocyte, HLA DR on CD33dim HLA DR+ CD11b-, CD25 on IgD- CD38br, CD80 on granulocyte, and CD39 on monocyte were negatively correlated with AS, serving as protective factors, while the remaining 11 were risk factors. HLA DR on CD14- CD16+ monocyte exhibited the highest significance level (*P* = 2.06*10^−5^) and remained significantly causal after FDR correction.

As shown in Fig. 5B, there were nominally significant causal relationships between AS and 9 immune phenotypes (*P* < 0.05). AS was negatively correlated with CCR2 on plasmacytoid Dendritic Cell, CCR2 on CD62L+ plasmacytoid Dendritic Cell, CD38 on CD20- B cell, CD19 on B cell, and CD19 on IgD+ CD24- B cell, while the remaining 4 were positively correlated. CD19 on B cell exhibited the highest significance level (*P* = 0.02). After FDR correction, no immune phenotype with significant causal association was found.

## Discussion

Through the systematic integration of large-scale summary GWAS datasets, our study provided a genetic viewpoint on the mechanisms underlying immune cell participation in the start and progression of Ankylosing spondylitis (AS). By incorporating two-sample MR techniques and using SNPs as instrumental factors, we were able to establish a relationship between the likelihood of acquiring AS and 22 different types of immune cells. The development of AS may lead to alterations in certain immune cells. Previous bioinformatics studies have indicated significant differences in naive CD4+ T cells, CD8+ T cells, and other immune cells between patients with ankylosing spondylitis and normal control group, closely associated with the onset and progression of AS [26–28]. Generally, AS is characterized by an increase in neutrophils, macrophages/monocytes, and synovial infiltration. When compared to healthy people, the blood cell counts of AS patients show higher platelet-to-lymphocyte ratio (PLR), neutrophil-to-lymphocyte ratio (NLR), and monocyte-to-lymphocyte ratio (MLR) compared to healthy individuals [29, 30]. Monitoring and restoring immune system function are crucial for the prompt identification and management of AS patients. We believe that the quantity, morphological parameters, and surface material expression associated with immune cells may play a crucial role in regulating the onset and progression of ankylosing spondylitis. Therefore, further research in this field is necessary.

Multiple studies have indicated the crucial role of T cells in the pathogenesis of AS. Our research findings suggest a close correlation between immune traits in the Maturation stages of T cell, Treg, and TBNK panels and ankylosing spondylitis. Firstly, the imbalance in the quantity and subpopulation proportions of these cells is observed. Cytotoxic cell gene expression is lost in AS patients due to a decrease in the absolute number of CD8+ T cells. Removing intracellular infections and cancerous cells is a major function of CD8+ T cells [31]. They guard against long-term immune system disorders and have a direct connection to the etiology of illnesses like ankylosing spondylitis (AS) and rheumatoid arthritis (RA) [32]. In our results, AS leads to a decrease in CD8+ Natural Killer T Absolute Count, CD8+ Natural Killer T %lymphocyte, CD8+ Natural Killer T %T cell, Terminally Differentiated CD8+ T cell %CD8+ T cell, CD28- CD8+ T cell %T cell, Central Memory CD8+ T cell %CD8+ T cell. CD8+ CD28− T cells are associated with greater degrees of joint restrictions [33]. Secondly, there is an imbalance in T-cell subset proportions. For example, Treg proportions are lower [34], while Th1/Th2 and Th17/Treg ratios are significantly higher than in normal populations [35]. Female AS patients exhibit upregulation of Treg cells and naive CD8 T cells [27]. Regardless of the maturity level of T cells, they may be influenced by the onset of AS, leading to a decrease in their numbers and symptoms associated with AS. Moreover, these cells’ distribution is affected: CD8 T cells tend to accumulate in inflamed joints of AS patients [36]; Additionally, there are changes in their surface receptor characteristics: after stimulation, peripheral blood monocytes in AS patients and CD8 T cells exhibit altered receptor characteristics, with significantly increased specificity clones for EBV and CMV [32]. HVEM on CD8+ T cells increases during the onset of AS. HVEM is a member of the TNF receptor family. Meanwhile, HLA DR+ CD4+ AC, CD8 on EM CD8br, CD8 on TD CD8br all increase the risk of developing AS. This suggests that one of the disease’s pathological characteristics is adaptive immunity [37]. Last but not least, patients with AS have aberrant cytokine release by T cells and subsets: CD4+ T cells secrete less IL-10. Conversely, CD8+ T cell production of IL-6[40] and two immunological mediators, IL-17A and interferon (IFN)-γ, rise dramatically [35].

Osteoclasts are a key component in the development of AS, and monocytes/macrophages are their precursors [38]. AS patients have lower numbers of monocytes/macrophages, and this is negatively correlated with the disease progression [39]. Patients with Spondyloarthritis (SpA) have considerably higher levels of CCR2 (Chemokine (C–C motif) receptor 2) M2 macrophages (CD163) in their peripheral blood (PB) and synovial biopsies, which are linked to AS disease activity [40]. Murine models have shown that mesenchymal stem cells from interphalangeal bones can secrete CCL2 (MCP1), which binds with CCR2/4 under mechanical stress [41]. Moreover, AS patients’ mesenchymal stem cells (MSCs) produce more CCL2 [42]. The CCL2/CCR2 axis not only participates in chemotaxis but also influences macrophage polarization. Macrophages polarize toward an inflammatory M1 phenotype when CCR2 is blocked [43]. Since that RA patients and antigen-induced arthritis (AIA) animal models both exhibit high levels of CCR2 expression and reactivity, neutrophil infiltration may also benefit from this axis. In the positive analysis, an increase in CCR2 on CD14+ CD16+ monocytes leads to a higher risk of developing AS. It is noteworthy that, in our analysis, the onset of AS leads to reduced CCR2 expression on plasmacytoid dendritic cells and CD62L+ plasmacytoid dendritic cells. This finding differs from previous research results. Dendritic cells (DCs) are essential for bridging innate and adaptive immunity [44], and are closely related to the induction and maintenance of SpA [45]. Plasmacytoid dendritic cells belong to the dendritic cell family, originating from bone marrow (BM), and have a plasma cell-like morphology. They can produce large amounts of type I/III interferons (IFN) and inflammatory cytokines after activation by pathogenic nucleic acids [46]. However, there is little research on the role and mechanism of CCR2 on pDCs in AS. Monocytes express the chemokine receptor CX3CR1, which is a crucial regulator of monocyte adhesion and migration [47]. The M2 phenotype is capable of identifying alternate activation subgroups of monocytes/macrophages and M2-like monocytes are classified as CD163, CX3CR1 and CD206 cells [48, 49]. According to Zhao et al.’s research [40] the M2 phenotype is the predominant phenotype of monocytes/macrophages in the peripheral blood and local tissues of late-stage AS patients, confirming that the pathological features of late-stage AS involve tissue repair and remodeling. Our results suggest that an increase in CX3CR1 on monocytes increases the risk of developing AS. In AS patients, CX3CR1 monocytes exhibit a distinct pro-inflammatory transcriptome and they play an active role in triggering the activation and expansion of ILC3, encouraging the prolonged pro-inflammatory condition in AS [50]. Therefore, CCR2 and CX3CR1 are generally upregulated in AS patients and play important roles in macrophage chemotaxis, polarization processes, and monocyte adhesion and migration.

The humoral immune response, specifically B cells, has received relatively less attention in the study of the pathogenesis of AS in the past few decades. The primary cause of this is the incapacity to establish the presence of common autoantibodies linked to AS. Nonetheless, there are signs point to the role of B cells in the pathophysiology of AS, including genetic variations related to B cell function, changes in the total number and distribution of B cell subpopulations, formation of (auto)antibodies, pathogenic B cell-related cytokines, and B cell infiltration in inflammatory sites susceptible to AS infection [51–53]. In patients with ankylosing spondylitis, most studies have found an increased frequency of circulating plasma cells (CD27hi/CD38hi cells) [51, 53]. Increased expression of many cell surface markers, such as CD86, CD38, and CD95, is linked to B cell activation [54, 55]. The onset of AS leads to an increase in CD20- CD38- B cell absolute count, CD20- CD38- B cell %lymphocyte, and CD20- CD38- B cell %B cell, while reducing CD38 on CD20- B cell. After FDR correction, AS still leads to an increase in CD20- B cell %lymphocyte and CD20- B cell %B cell. Moreover, in the positive direction, IgD- CD38dim %lymphocyte and CD19 on IgD+ are associated with an increased risk of AS. The roles of these B cells in the onset and development of AS provide a theoretical basis for B cell-targeted therapies for AS. In various immune-mediated diseases, the most famous and widely studied anti-B cell therapy is the use of B cell-depleting biologics such as rituximab, which targets CD20-positive B cells for treatment [56]. However, there have been few observational studies on the efficacy of rituximab in treating AS, and the sample sizes involved in these studies have been limited. Additionally, there have been no placebo-controlled trials using rituximab to treat ankylosing spondylitis. However, B cell depletion seems to be beneficial for AS patients who are unresponsive to TNFα inhibitors [57, 58]. It will take more investigation with a bigger group of clearly characterized ankylosing spondylitis patients to identify which people might most benefit from anti-B cell therapy. Subsequent studies should also focus on exploring the mechanisms of these benefits. Our study provides a theoretical foundation for the development of precise B cell depletion targeted therapy for AS treatment in the future.

Osteolytic destruction and ossification are two pathogenic features of AS, which are associated by immune system abnormalities. In the study of AS pathogenesis, the control and interaction between the skeletal and immune systems have gained a lot of attention. Our results show that immune cells may have a causative role in ankylosing spondylitis. This guides the development of novel medications and offers critical support for clinical prognosis and therapy decisions. Nonetheless, the pathophysiology of AS is complicated, and the various immune cell types implicated in AS exhibit considerable clinical variability. Singular treatments often do not yield satisfactory results. Therefore, further research is required to comprehend how innate immune cells interact with one another in AS patients as well as how innate and adaptive immune cells interact.

Undeniably, our study undoubtedly has certain shortcomings. First, we chose the largest GWAS summary datasets for AS samples and immunological characteristics. These two sets of data, however, are from distinct research, which might add inaccuracies because of variations in sample size, quality control techniques, and ethnicity. Secondly, individual-level data was absent from our analysis, which relied on summary-level datasets. Therefore, this study was unable to conduct further stratified analyses for the traits of interest (such as gender, age, etc.). Thirdly, despite undergoing FDR multiple corrections, the lenient threshold for SNP selection criterion due to limited sample size might result in a certain degree of false positives. Nevertheless, this also opens up the possibility for a comprehensive assessment of the interactions between the immune system and ankylosing spondylitis. Besides, we recognize that the linkage disequilibrium (LD) between HLA genes [59], particularly HLA-B27 [60], presenting a potential limitation to our findings. Further fine-mapping or conditional analyses would be necessary to disentangle the roles of HLA genes and nearby linked loci in ankylosing spondylitis pathogenesis. This limitation should be considered when interpreting the causal relationships identified in this study.

## Conclusions

In conclusion, we think that the findings of our investigation offer fresh perspectives on the immunology of AS etiology. To investigate the possible processes behind the discovered immunological characteristics and their correlation with AS risk, more experimental study should be carried out.

## Electronic supplementary material

Below is the link to the electronic supplementary material.

Supplementary Material 1

Supplementary Material 2

Supplementary Material 3

Supplementary Material 4

## Data Availability

To request data, please contact the respective author.

## Abbreviations

AS: Ankylosing spondylitis
GWAS: Genome-wide association studies
IL: Interleukin
MR: Mendelian Randomization
SNP: Single Nucleotide Polymorphisms
IVs: Instrumental Variables
cDC: Conventional dendritic cells
AC: Absolute cell
MP: Morphological parameter
MFI: Median fluorescence intensity
RC: Relative cell
IVW: Inverse Variance Weighted
MR-PRESSO: Mendelian Randomization Pleiotropy Residual Sum and Outlier
PLR: Platelet-to-lymphocyte ratio
NLR: Neutrophil-to-lymphocyte ratio
MLR: Monocyte-to-lymphocyte ratio
RA: Rheumatoid arthritis
IFN: Interferon
SpA: Spondyloarthritis
MSCs: Mesenchymal stem cells
