# Supplementary material for: Investigating the causal relationship between immune factors and ankylosing spondylitis: insights from a Mendelian Randomization study
Source: Adv Rheumatol. 2024 Dec 18;64:89. doi: 10.1186/s42358-024-00428-1 (PMC13488627; doi:10.1186/s42358-024-00428-1)
Supplement: Supplementary file 1 [file 42358_2024_428_MOESM1_ESM.docx]

Supplementary Figure1: Phenotypes and Panel Classification of Immune Cells Associated with AS Onset

Supplementary Figure2: Scatter Plots and Funnel Plots of Phenotypes that Remain Positive After FDR Correction

Supplementary Table 1: Detailed information about the panels and phenotype categories for each immune phenotype

Supplementary Table 2: Detailed information on all positive result SNPs

Supplementary Table 3: Diverse Mendelian randomization (MR) results for 22 immune features causally associated with AS

Supplementary Table 4: Diverse Mendelian randomization (MR) results for 34 immune features causally affected by AS

Supplementary Table 5: The heterogeneity and pleiotropy analysis results for positive outcomes in the forward analysis.

Supplementary Table 6: The heterogeneity and pleiotropy analysis results for positive outcomes in the reverse analysis.
